# Unbalanced visual cues do not affect search precision at the nest in desert ants (*Cataglyphis nodus*)

**DOI:** 10.3758/s13420-023-00613-0

**Published:** 2023-11-20

**Authors:** Patrick Schultheiss

**Affiliations:** Behavioral Physiology and Sociobiology, University of Würzburg, Am Hubland, 97074 Würzburg, Germany

**Keywords:** Area-restricted search, Formicidae, Panorama, Systematic search, Visual navigation

## Abstract

Desert ant foragers are well known for their visual navigation abilities, relying on visual cues in the environment to find their way along routes back to the nest. If the inconspicuous nest entrance is missed, ants engage in a highly structured systematic search until it is discovered. Searching ants continue to be guided by visual cues surrounding the nest, from which they derive a location estimate. The precision level of this estimate depends on the information content of the nest panorama. This study examines whether search precision is also affected by the directional distribution of visual information. The systematic searching behavior of ants is examined under laboratory settings. Two different visual scenarios are compared – a balanced one where visual information is evenly distributed, and an unbalanced one where all visual information is located on one side of an experimental arena. The identity and number of visual objects is similar over both conditions. The ants search with comparable precision in both conditions. Even in the visually unbalanced condition, searches are characterized by balanced precision on both sides of the arena. This finding lends support to the idea that ants memorize the visual scenery at the nest as panoramic views from different locations. A searching ant is thus able to estimate its location with equal precision in all directions, leading to symmetrical search paths.

**Supplementary Information:**

The online version contains supplementary material available at 10.3758/s13420-023-00613-0.

## Introduction

Ants are highly skilled navigators. As central place foragers, they leave the nest to forage for food, but then must find their way back home to share the collected food with their nestmates. To this end, they use a range of different environmental cues and sensory modalities, such as olfaction and vision. Over the years, thermophilic desert ants have become well-studied organisms for understanding visual navigation on a mechanistic level, and scientists such as Ken Cheng and Rüdiger Wehner have devoted large chunks of their professional careers to this subject (Cheng, 2022; Cheng & Freas, 2015; Cheng et al., 2014; Wehner, 2019; Wehner, 2020). In desert ant navigation, olfaction plays a subordinate role because chemical trails – which are used for orientation in many other species of ants – do not persist long enough on the hot desert floor to be a navigational aid. Instead, these ants navigate predominantly by visual means, with an entire toolkit of different strategies at their disposal (Wehner, 2008). Two main strategies stand out: path integration and view-based guidance mechanisms (Schultheiss et al., 2020). Ants often use these two strategies in concert.

In path integration, the ant keeps track of its heading directions using celestial compass cues, most prominently the polarized pattern of sunlight (Fent, 1986); at the same time its walked distance is tracked by an odometric system (Wittlinger et al., 2006). At any point in its journey, the ant is able to integrate this direction and distance information to derive a homeward-pointing vector (Wehner & Srinivasan, 2003). Most impressively, path integration is used by the North African desert ant *Cataglyphis fortis*, which inhabits featureless dry saltpans. However, most ant species live in habitats with prominent visual features available for navigation. This is the case for the species investigated here (the Mediterranean desert ant *Cataglyphis nodus*), which relies strongly on view-based guidance mechanisms (Fleischmann et al., 2018). These ants acquire rich visual memories of places and routes, and later retrieve these memories for navigational guidance (Cheng et al., 2009). The visual cues that are used for guidance are terrestrial in nature and often referred to as “landmarks.” However, as ants make use of all manner of terrestrial visual features, such as the shape of the skyline rather than individual landmarks (Graham & Cheng, 2009; Wystrach et al., 2011), the term “view” is preferable. The visual memory is thought to be organized in more or less distinct views of the environment at different locations – snapshots (Judd & Collett, 1998) – though it remains unclear exactly what kind of information may be included in such views (Dittmar et al., 2010). Navigational guidance is likely derived from views in a matching procedure, in which the currently perceived view is compared to a memorized view; the ant then moves in a direction where the visual match improves (Zeil, 2012).

The most important view that foraging ants need to memorize is the view around the nest entrance. If the inconspicuous entrance is not found, the forager is in danger of roasting in the desert heat and the rest of the colony will not get the food. For this reason, ants that emerge from the nest to start their foraging career engage in learning walks for the first two days. These are short, looping excursions, where the ant stays close to the entrance and forms a robust visual memory of the nest surround. The formation of visual memory is thought to occur at instances where the ant makes brief pirouettes or stop-and-scan motions, and looks back toward the nest entrance (Deeti & Cheng, 2021; Deeti et al., 2023; Fleischmann et al., 2017; Zeil & Fleischmann, 2019).

The success of all navigation strategies depends on the ultimate discovery of the goal. For a returning forager this is not a trivial task as the nest entrance can be extremely inconspicuous, and it engages in a highly structured systematic search until it is found (Schultheiss et al., 2015). The initial starting point of the search is the location estimate from previous guidance routines. The following search path then consists of a systematic series of loops in which the animal explores the vicinity and repeatedly returns to the starting point. It contains some elements of spiral movements (Müller & Wehner, 1994) and is structured in a way that reduces overlap and ensures exploration in all directions (Schultheiss et al., 2015; Wehner & Srinivasan, 1981). However, this searching behavior also remains highly flexible: the navigation processes that initially led the animal to the starting point continue to operate throughout the search and can further shape its structure.

All navigational strategies can only provide location estimates with a certain level of accuracy and a certain level of precision. Accuracy describes the proximity of the estimate to the actual target location, and precision describes the reliability of that estimate. The search paths of ants are adapted to accommodate both these measures: the center of search reflects the estimate accuracy, while the overall size of the path reflects the estimate precision (Schultheiss et al., 2015). Searches in featureless environments are much larger than searches in familiar visual environments (Wehner et al., 1996; Wehner & Srinivasan, 1981), highlighting the low overall precision of path integration guidance compared to view guidance strategies. Search size is further increased when the path integration mechanism suffers from reduced certainty (Merkle et al., 2006; Schultheiss & Cheng, 2011). In view-based navigation, diminished precision may be the result of unfamiliarity with the view (Schultheiss et al., 2016) or of reduced information content of the view (Schultheiss et al., 2013). In the latter study, this was investigated through a comparison of searching behavior in a visually enriched environment with a visually impoverished environment where the overall number of visual objects was reduced. The impoverished setting provided less visual information to navigate by, resulting in reduced precision of the positional estimate and an increase in search size. Yet, it remains unclear so far how searching behavior would be affected by a strongly unbalanced visual environment, where all visual information is contained in only one half of the panorama. A searching ant may be able to navigate with greater precision on the visually rich side than on the impoverished side.

The current study investigates this question in the desert ant *Cataglyphis nodus* Brullé, 1833, which is known to have excellent visual navigation abilities (Fleischmann et al., 2017; Rössler, 2019). I focus on the nest-searching behavior, as the motivation of forager ants to return home is extremely high. Through controlled manipulation of the visual environment, I aim to examine whether search precision is determined by the overall information content of the view around the nest, or if an unbalanced view results in unbalanced search precision.

## Methods

Experiments were performed on the desert ant species *Cataglyphis nodus*. These ants inhabit the Eastern Mediterranean regions, where they occupy the ecological niche of a thermophilic, diurnal scavenger. Their natural habitat is well vegetated and visually complex, and experienced foragers strongly rely on these visual cues (Fleischmann et al., 2018). Colonies of these ants were housed at the University of Würzburg in a climate-controlled room (27 °C, 40% humidity), with access to small foraging areas where honey water, mealworms, and water were provided *ad libitum*. The foraging areas were kept under a 12/12-h light-dark cycle, while the colonies themselves were kept in darkness. Two different colonies of ants were used in this study.

A queenright colony with several hundred worker ants was moved to a separate, smaller dark climate chamber. A pipe connected this chamber to the center of a larger indoor foraging arena (1.76 × 1.76 m) from below, thus simulating a natural nest entrance with a tunnel leading to the underground nest. A low barrier (10 cm high) at the perimeter of the arena ensured that the ants could not escape while allowing views of the surrounding experimental room. Food items (mealworms and cookie crumbs) were provided at the edge of the arena, always at the same location. The arena was evenly lighted from above by two 58 W fluorescent tubes, and the temperature of the room was kept at 25–27 °C. Foraging ants were free to enter and explore the arena, and to learn the spatial layout of the visual environment for at least 2 days before testing.

**Fig. 1:**
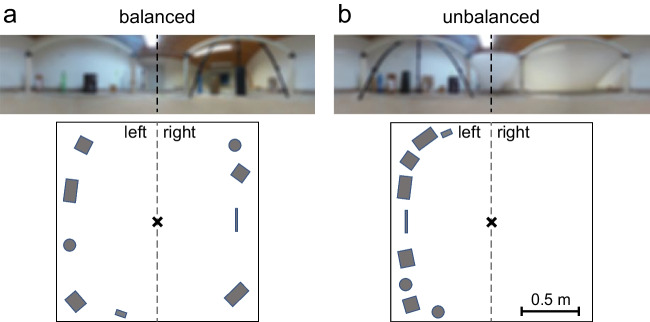
Overview of the setup. (**a**) Shows the balanced visual condition, and (**b**) shows the unbalanced visual condition. At the top are panoramic views from the nest entrance at the center of the arena (resolution reduced to 3° per pixel to approximate ant vision), below are schematics of the distribution of the visual cues (objects) around the experimental arena in top view, drawn to scale. The cross marks the location of the nest entrance, and the dashed line shows the separation of the arena into left and right sides for later analysis

Two different visual environments were implemented by placing objects around the edge of the arena (object height: 15–50 cm). In the first condition, the objects were distributed evenly around the edge, so that ants inside the arena would be faced with a reasonably balanced visual panorama. Visual cues from the experimental room contributed to the panorama, especially on the right side (Fig. 1a). In the second condition, the same objects were used but now placed on the left side of the arena. A white cotton sheet was hung at the right side to further occlude visual cues from outside the arena, greatly reducing the visual information content of this side. Overall, this manipulation resulted in a very unbalanced visual panorama (Fig. 1b). In each condition, ants were left to explore and experience the arena for at least 2 days before testing, ensuring that they had become familiar with the spatial layout of the visual environment. Different groups of ants were tested in the two visual conditions (balanced condition: Nest 1 *n* = 16, Nest 2 *n* = 21, combined *n* = 37; unbalanced condition: Nest 1 *n* = 17, Nest 2 *n* = 12, combined *n* = 29).

Inside the arena, foraging ants would pick up a food item and carry it back into the entrance at the center. These foragers would then often shuttle back and forth between the food and the nest entrance, repeatedly collecting food items for the rest of the colony. To study the systematic searching behavior of these ants, a small wooden board was placed over the nest entrance when a foraging ant picked up a food item and marched back towards the entrance. The edges of the board were covered with fine sand to smooth out the surface. With the entrance and its potential nest-associated odor cues blocked by the board, the ant embarked on its systematic searching behavior, which was filmed from above with a digital camera (GoPro Hero 4 Black at 24 fps, with ultra-wide field-of-view and a resolution of ≥ 1,080 pixels). The beginning of the search was defined as the point where the returning ant performed its first obvious turn (> 60°). Only ants that carried food items were recorded as this ensured high motivation for homing, and each ant was filmed for 2 min or longer, depending on their walking speed. Occasionally, other ants were simultaneously present in the arena with the focal ant (up to five individuals). Recorded ants were marked on their gaster with a spot of non-toxic acrylic paint, so as not to record the same individual again.

The videos were further processed to remove fisheye distortion (GoPro Studio software, version 2.5.11), converted to image sequences at 2 fps and 720-pixel resolution (OpenShot Video Editor software, version 2.5.1), and the path trajectory of the searching ant digitized (Fiji ImageJ software, version Madison with Manual Tracking plugin). All search paths were truncated at 8.5 m length to ensure equal amounts of data from different individuals. For each ant, the center of search was calculated as the median position of all digitized points of the path. To quantify the size of the search – which reflects search precision – the median distance of all digitized points from the nest entrance was calculated, because this is the target of the searching ant. The two ant colonies that were recorded did not differ in the measure of search size, and the data were thus pooled within conditions (Mann-Whitney *U* tests: balanced condition, *U* = 83, *p* = .08; unbalanced condition, *U* = 74, *p* = .23). In further statistical tests, I applied Bonferroni corrections when performing multiple comparisons between different subsets of the same dataset.

## Results

**Fig. 2:**
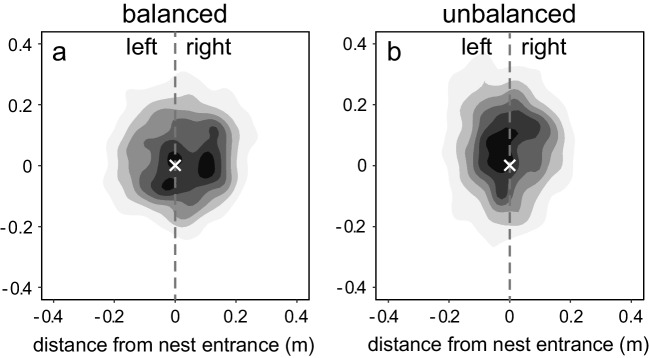
Ant search densities in top view, with the occluded nest entrance located at the zero position at the center of each panel. (**a**) Shows the search in the balanced visual condition (n = 37), and (**b**) shows the search in the unbalanced visual condition (n = 29). Black marks the highest search densities, and white the lowest densities. The small white cross marks the (covered) nest entrance. While the orientation is the same as in Fig. 1 (lower panels), the scale is different here

During their search for the nest entrance, the ants displayed typical, highly structured systematic search patterns. Their paths consisted of a series of loops, and they repeatedly returned close to the nest entrance. In both conditions, the search density profiles have the highest values in the vicinity of the nest entrance (Fig. 2), showing that this area was investigated most intensely by the searching ants. There is slight variation in search accuracy between individuals, with the center of search (the median position of all digitized path points) of individual ants located on average about 10 cm distant from the exact nest entrance (balanced condition, *n* = 37, *M* = 11.4 cm, *SD* = 6.8 cm; unbalanced condition, *n* = 29, *M* = 12.8 cm, *SD* = 4.9 cm). However, this navigational error of individual ants is small, with no difference between the two experimental groups evident (*t*-test: *t*(64) = 0.93, *p* = .36).

**Fig. 3:**
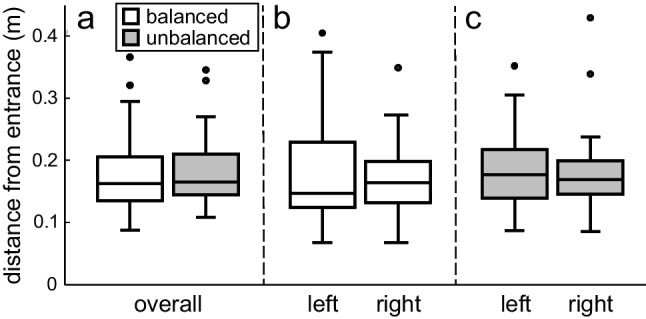
Search size (median distance from the nest entrance) under the two different visual conditions, the balanced condition is shown in white and the unbalanced condition in gray. (**a**) Comparison of overall search size, (**b**) comparison of the left and right sides within the balanced condition, and (**c**) comparison of the left and right sides within the unbalanced condition. Statistical results indicate that none of the group comparisons are significant

In terms of search precision – the size of the inspected area – the ants performed surprisingly well. The search density profiles in Fig. 2 show that the area of more concentrated searching is confined to only 0.5 × 0.5 m^2^ in both conditions. Searching ants did sometimes venture out into more peripheral regions of the arena (its closest edge was at 0.88 m distance from the entrance), but not sufficiently to show up in the density profile of the entire group. In terms of overall size, the searches under the two visual conditions do not appear very different from each other (Fig. 2). This impression was confirmed by formal statistics, using each ant’s median distance from the nest entrance as its measure of search size (Fig. 3a). The two groups are not significantly different from each other in terms of this measure (*U* = 503, *p* = .67).

In the balanced visual condition, where the visual objects were evenly distributed around the arena edge, the search effort is symmetrical around the nest entrance position (Fig. 2a). Measuring the median distance from the nest entrance, there is no significant difference between the left and right sides (Wilcoxon signed ranks test: *Z* = 0.45, *p* = .67; Fig. 3b). For the unbalanced condition, where all visual objects were placed on the left side of the arena, the picture is much the same (Fig. 2b). Here too, there is no significant difference between the left and right sides of the search (*Z* = 0.81, *p* = .43; Fig. 3c).

## Discussion

This study investigates the systematic searching behavior of desert ants in an indoor laboratory setting – to my knowledge for the first time. The ants performed well under these settings, with their search paths displaying all the hallmarks of systematic searches under natural conditions – such as search loops and frequent returns to the nest entrance (Schultheiss et al., 2015). This is an encouraging finding for future studies of this kind in laboratory settings. The ants’ searches are surprisingly tight – i.e., cover a small area around the nest entrance – which demonstrates that they are strongly guided by the familiar visual cues in their environment. In fact, they are considerably tighter than naturally observed searches (Fleischmann et al., 2018), but then the experimental setup simulates a visual environment that is unusually compact. In their natural habitat of Mediterranean shrubland or open forest, it is very uncommon to have visual objects in such close proximity to the nest (personal observation). Rather, the small search size showcases the extreme flexibility of this behavior, which is adapted to the specific visual context. In featureless salt-pans – devoid of terrestrial visual cues but providing celestial cues – the nest searches of *Cataglyphis* foragers are considerably larger, covering several square meters (Wehner & Srinivasan, 1981).

Desert ants are guided by familiar visual cues during their systematic searching behavior (Schultheiss et al., 2013). Here, I investigate whether the spatial distribution of such visual cues would affect their search precision, specifically whether an unbalanced visual panorama would lead to searches with skewed precision. The results indicate that this is not the case. Regardless of whether the visual setting was balanced or unbalanced, the precision of the ants’ searches remained unaffected and balanced.

It is important to note here that the number and identity of close visual objects around the arena edge was not changed between the two visual conditions; the only modification was in the spatial (azimuthal) distribution of these objects. Visual cues outside the arena (including the door and walls of the experimental room) remained fully visible in the balanced condition but were asymmetrically obscured by a white sheet in the unbalanced condition, further increasing the directional imbalance of visual information. So, while the two conditions differed greatly in their directional balance of visual information, their total information content of the entire visual panorama, as seen from the nest entrance, is similar (see Fig. S1 in the Online Supplemental Material). Since the searches in both visual conditions were characterized by similar levels of precision, this could be seen as an indication that searching ants rely on entire panoramic views for guidance, as they appear to do in directional navigation (Buehlmann et al., 2016; Graham & Cheng, 2009).

Moreover, a searching ant will perceive more than just the one view from the nest entrance. As the ant moves around the arena, the subtended retinal angles and apparent sizes of visual objects will change continually, and the dynamics of visual change will be subtly different in the two conditions. The information content of such a visual landscape can be well understood through an image difference function, where new views from different locations around the target are compared to a reference image at the target location itself (Zeil et al., 2003). Quantifying image differences (e.g., pixel differences) of these image pairs and mapping them onto the positions of the new images leads to a translational image difference function of the landscape. At the target itself the value is always zero (where the reference image is compared to itself), and image difference values increase from there in a funnel-shaped manner as one moves away from the target. Information-rich environments have a steep function, whereas information-sparse environments have a shallower function (Schultheiss et al., 2013). It has been suggested that insects, and also rats, may be able to navigate towards a close target by descending the gradient of such a difference function (Cheung et al., 2008; Zeil et al., 2003).

In the current study, the experimental manipulations have created two visual environments in which the translational image difference functions will be characterized by different shapes: mostly balanced in the balanced visual setting, and lop-sided in the unbalanced setting with a steeper slope towards the visual cues and a shallower slope towards the other half of the arena. However, ants in the unbalanced setting do not search in a lop-sided manner. Thus, a visual navigation strategy based on simple gradient descent towards a single reference view does not entirely capture the actual behavior of searching ants. An alternative interpretation would be that these ants memorize a series of reference views at different locations close to the nest entrance. This squares well with our current understanding of how ants acquire nest views during their first learning walks. During such walks, the ants perform multiple short stops or pirouettes at different locations and look back towards the nest entrance (Deeti & Cheng, 2021; Fleischmann et al., 2017). If indeed view memories are stored at these short stops, as has been suggested (Zeil & Fleischmann, 2019), this would result in a visual memory of the nest area that is composed of a series of views rather than a single one. In combination with the idea that such memorized views are truly panoramic (see above), even visually unbalanced environments would be memorized in a balanced fashion. Thus, a returning ant that is searching for its nest entrance will be able to estimate its location with equal precision in all directions, leading to symmetrical search paths. Robustly symmetrical searching behavior should prove particularly beneficial under natural conditions, where relevant visual information (e.g., vegetation) is unlikely to be distributed in a balanced manner.

### Supplementary information

ESM 1 (PDF 108 kb)
