# Supplementary material for: Unbalanced visual cues do not affect search precision at the nest in desert ants (Cataglyphis nodus)
Source: Learn Behav. 2023 Nov 20;52(1):85–91. doi: 10.3758/s13420-023-00613-0 (PMC10923989; doi:10.3758/s13420-023-00613-0)
Supplement: Supplementary file 1 — (PDF 108 kb) [file 13420_2023_613_MOESM1_ESM.pdf]

Supplementary Material

for

Unbalanced visual cues do not affect search precision at the nest in desert ants (*Cataglyphis nodus*)

Patrick Schultheiss

ppschultheiss@gmail.com

Figure S1

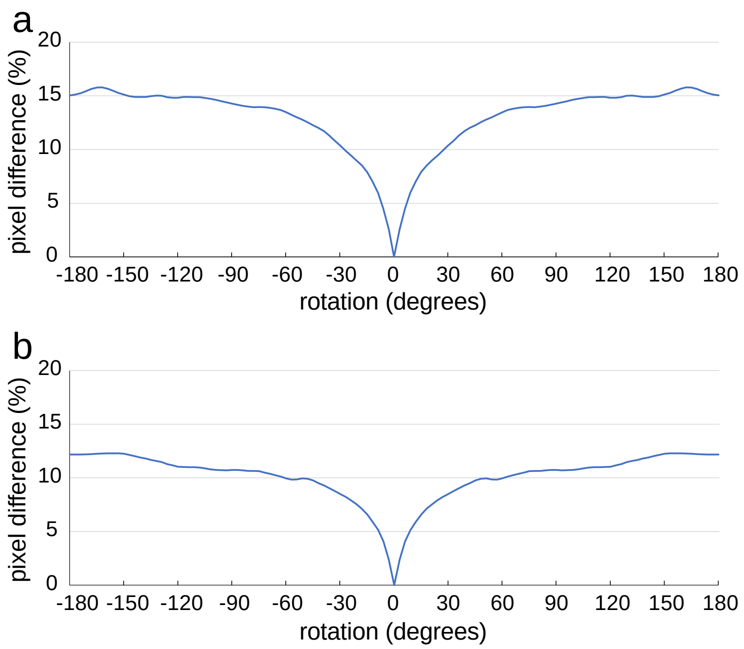

Figure S1: Rotational image difference functions of the view from the nest entrance in the two different visual conditions. **a)** Balanced visual condition, **b)** unbalanced visual condition. Images were converted to grayscale with a resolution of 3 degrees.
